# The Role of Immunocytochemical Markers to Differentiate Primary from Secondary Neoplastic Hepatic Masses: A Diagnostic Challenge on Cytology

**DOI:** 10.5146/tjpath.2021.01527

**Published:** 2021-09-15

**Authors:** Jenna B Bhattacharya, Shyam Lata Jain, Subbarayan Devi

**Affiliations:** Department of Pathology, Lady Hardinge Medical College, New Delhi, India; Maulana Azad Medical College & Hospital, New Delhi, India; Chettinad Hospital and Research Institute, Chennai, India

**Keywords:** Hepatic neoplasm, Liver metastases, Immunocytochemistry, Cytology, HepPar1

## Abstract

***Objective:*** It is challenging and difficult to differentiate primary from metastatic hepatic masses solely on cytology. The present study aimed to correlate cytomorphological spectrum of hepatic masses with immunocytochemical markers to differentiate primary from metastases in liver.

***Material and Method:*** The present study comprised of 30 clinico-radiologically suspicious cases of neoplastic hepatic masses. Ultrasound-guided fine needle aspiration smears and cell blocks were prepared as per standard technique; two of the smears were air-dried and Giemsa stained to study cytomorphological features. A panel of markers (HepPar-1, CD 10, CK7, CK19, CD34, and MOC-31) were studied both in smears and cell blocks.

***Results:*** Cytomorphological features on smears were evaluated and correlated with immunocytochemistry in all cases; the final diagnosis was: Hepatocellular carcinoma (n=7), cholangiocarcinoma (n=2), hepatoblastoma (n=1) and metastatic carcinoma (n=20). HepPar-1, CD10 and CD34 demonstrated 86%, 72%, 86% sensitivity and 100% specificity respectively for hepatocellular carcinoma; CK7&CK19 showed 100% sensitivity for cholangiocarcinoma, MOC 31 showed 90% sensitivity and 100% specificity for metastatic carcinoma.

***Conclusion:*** The present study recommends a panel of minimum three markers (HepPar-1, CD10, and MOC-31) which were helpful to differentiate hepatocellular carcinoma from metastatic carcinoma that was a major diagnostic challenge solely on cytomorphology. Correlating cytomorphology with these three markers, 100% of the cases could be diagnosed as primary malignancy and distinguished accurately from metastatic carcinoma.

The distinction between primary and metastases in liver on cytomorphology alone has been challenging and a matter of much debate. Though some cytomorphological features help to differentiate hepatocellular carcinoma (HCC) from metastatic carcinoma (MC), the distinction between the two still remains a challenge in case of poorly differentiated tumors. A cytopathologist also faces diagnostic challenges to differentiate benign/reactive hepatocytes from well-differentiated HCC (WDHCC). Hence, a panel of immunocytochemical markers (ICC) is advised for the final diagnosis in challenging cases. Various studies were conducted to assess the role of ICC in hepatic masses, using a single marker or panel of markers when a diagnosis on cytomorphology alone was difficult (1–13). Also, since the prognosis and treatment of both HCC and MC are significantly different, ancillary techniques for correct diagnosis and further follow up are mandatory. Human hepatocyte antibody-1 (HepPar-1) has been reported as a highly specific marker for hepatocytic differentiation with high sensitivity (90%). (4,5,11).

Despite the availability of many markers, no single marker is 100% specific and sensitive for either HCC or MC so far (5–16). Hence, the present study aims to evaluate a panel of markers (HepPar-1, CD10, CK7, CK19, CD34, MOC31) along with cytomorphology to differentiate HCC from MC.

## MATERIAL and METHODS

This prospective study was conducted in the departments of pathology and gastroenterology surgery after obtaining ethical clearance and written informed consent from the patients. 30 clinico-radiologically suspected cases of neoplastic hepatic mass lesions were aspirated. Patients with abnormal coagulation profile, vascular lesions and infective cysts were excluded. Relevant clinic-radiological and serological findings were recorded. The study was approved by the Institutional Ethics Committee (Date: 04.08.2011, Ref. No: 11/1EC/MAMC/2011/119)

USG/CT-guided FNAC was performed as per standard technique using a 23/24 gauge needle. Multiple areas were aspirated for an adequate and representative sample. Two smears were air-dried and Giemsa stained to study morphological features and the rest was preserved at 0°C for ICC. The remaining aspirate was processed for the cell block (CB). To prepare CBs, the hemorrhagic aspirate was allowed to clot for 1-3 hrs, fixed in 10% buffered formalin, and processed further as routine Hematoxylin & Eosin (H&E) stained histological sections for cyto-histological correlation. ICC was performed on fresh smears (n=16) and on both smears and CB (n=14). For ICC, the smears were fixed in a mixture of cold acetone and methanol (1:1) for 5 minutes; and for CBs, 3-5µ thick sections were heated to deparaffinize followed by changes in xylene, graded alcohol and hydration in water. All steps were carried out in a moist and humid chamber throughout the procedure. The primary antibodies (Ab) used were as follows: HepPar-1 (monoclonal mouse Ab, DAKO, clone OCHIE5), CD10 (monoclonal mouse Ab, DAKO), MOC31 (anti-mouse Ab, BioSB, clone BerEp4), CK7 (monoclonal mouse Ab, DAKO, Flex, clone OV-TL) and CK19 (monoclonal mouse Ab, Flex DAKO, clone RCK 108), CD34 (Skytek, clone 4C4.9). The Biogenex Super Sensitive Polymer-HRP Detection System was used in conjunction with rabbit/mouse IgG Primary Abs. DAB (diaminobenzidine) substrate for peroxidase was used as an enzyme label forming a stable brown end product at the site of the antigen (Ag). A fresh citrate buffer was prepared with every set of staining and used for Ag retrieval wherever required. Hematoxylin was used as a counter stain. Positive and negative controls were run with every batch.

Two authors independently evaluated every case. Granular cytoplasmic positivity for HepPar-1; canalicular, cytoplasmic and membranous stain for CD10; endothelial cell stain for CD34; membranous/membranous and cytoplasmic stain for MOC31; and cytoplasmic stain pattern for CK7& CK19 were considered significant and positive. Statistical analysis was done using the Chi square test and Fisher’s exact test using SPSS software.

## RESULTS

**Figure 1:**
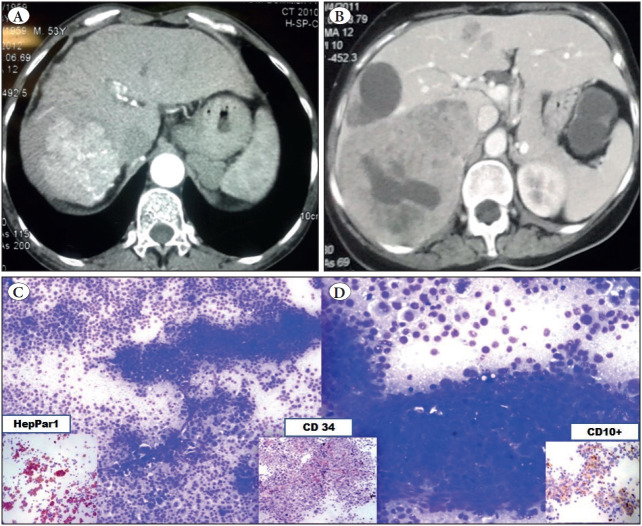
**A)** CT image showing a single spaceoccupying lesion. **B)** CT image showing multiple space-occupying lesions suggesting metastases. **C)** FNA smears showing atypical cells with perivascular arrangement, scattered naked nuclei (Giemsa; x400). **D)** Well-differentiated HCC showing trabecular arrangement (Giemsa; x400). Figure insets: HepPar-1 showing strong granular cytoplasmic positivity (ICC; x400). CD10 showing strong granular cytoplasmic positivity (ICC; x400). CD34 showing strong grade 3 positivity (ICC; x400).

Of the 30 patients included in the study, 17 were male (56.6%) and 13 female (43.3%), with a male to female ratio of 1.3:1. The patients ranged in age from <1 to >80 years (mean age-48.03 ±1.73 years). The most common radiological finding was a single space-occupying lesion (SOL) in cases of HCC and multiple SOLs in cases of metastases (Figure 1).

The cytomorphological features studied encompassed cellularity, pattern of cell arrangement, cytoplasmic and nuclear details and the background. Based on these features and correlating with ICC, the final diagnosis was HCC (n=7), CC (n=2), HB (n=1) and MC (n=20) from various sites (Table 1).

**Table 1:** Final diagnosis on cytology and immunocytochemistry

**Category**		**No. of cases (n=30)**
HCC		7
HB		1
CC		2
Metastatic Adenocarcinoma	Gall Bladder	2
	Colon	5
	Stomach	4
	Ovary	4
	Unknown primary	2
	Adenocarcinoma with NE differentiation	1
Squamous cell carcinoma (primary lung)		1
Small cell carcinoma (lung)		1

**HCC:** Hepatocellular carcinoma, **HB:** Hepatoblastoma, **CC:** Cholangiocarcinoma

**Figure 2:**
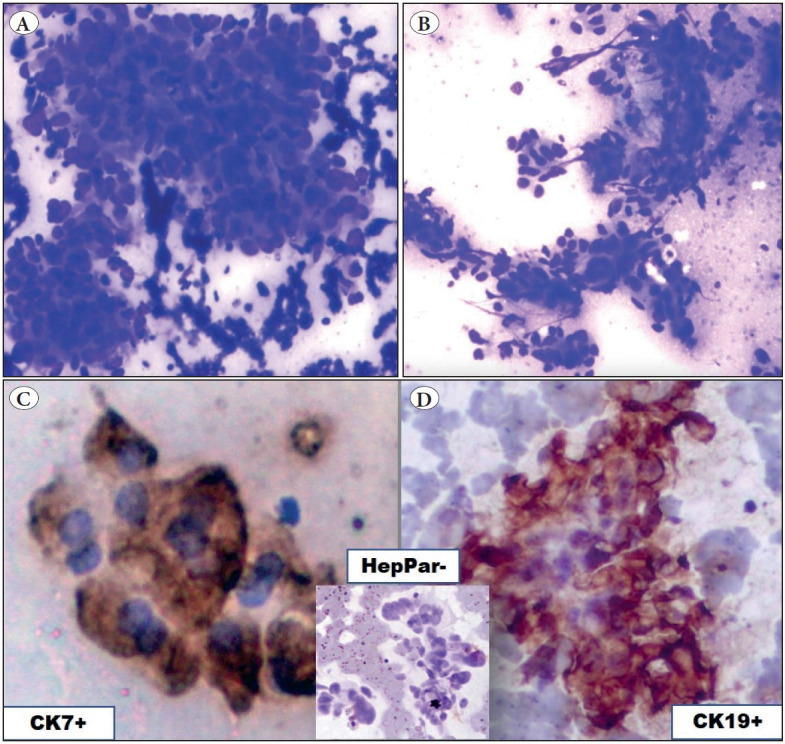
**A-B)** Cholangiocarcinoma showing atypical columnar cells in an acinar pattern with a fibrotic background (Giemsa; x400). **C)** CK7 showing strong cytoplasmic positivity in tumor cells (ICC; x400). **D)** CK19 showing strong cytoplasmic positivity in tumor cells (ICC; x400). (Inset: Tumor cells negative for Hep Par 1).

The following cytological features were useful in the diagnosis of HCC: clusters of tumor cells surrounded by capillaries (peri-sinusoidal pattern) and clusters with transgressing capillaries (transgressing/centri-sinusoidal pattern), bile pigment in tumor cells, intranuclear inclusions (INI), prominent macronucleoli, and atypical stripped nuclei in the background (Figure 1). Smears of CC showed a typical smear pattern of adenocarcinomas (AC) with clusters of columnar epithelial cells in an acinar pattern (Figure 2). Initially these cases were suggestive of a metastatic AC; however, only after the panel of ICC could they be correctly diagnosed as CC since both CK7 and CK19 were positive (Figure 2) and the rest of the markers were negative ruling out primary HCC/metastases even though the clinical diagnosis was suggestive of MC (Table 2).

**Table 2:** Immunocytochemical profile in various hepatic malignancies

**ICC markers**	**Hepatocellular carcinoma**	**Cholangio- carcinoma**	**Metastasis**
HepPar-1	6/7 (86%)	0/2 (0%)	0/20 (0%)
CD 10	5/ 7 (71.4%)	0/2 (0%)	0/20 (0%)
CD 34	6/7 (86%)	0/2 (0%)	0/20 (0%)
CK7	0/7 (0%)	2/2 (100%)	2/20 (10%)
CK19	0/7 (0%)	2/2 (100%)	5/20 (25%)
MOC 31	0/7 (0%)	2/2(100%)	18/20 (90%)

**ICC:** Immunocytochemistry

**Figure 3:**
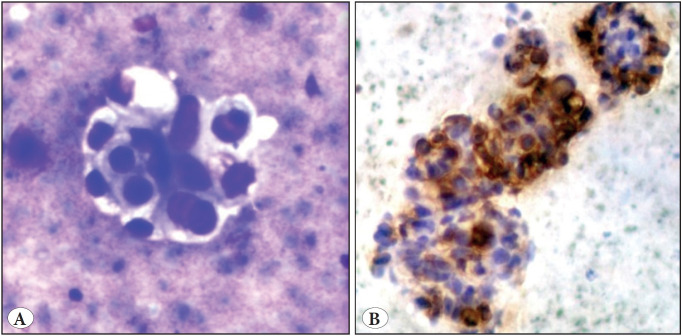
Metastatic adenocarcinoma. **A)** Atypical cells with intracytoplasmic mucin in a necrotic background (Giemsa; x400). **B)** MOC31 showing strong membranous accentuation in A B tumor cells (ICC; x400).

The MC group showed atypical cells with attempted acinar/papillary pattern, necrosis, and a mucinous and/inflammatory background. The important feature was the presence of benign hepatocytes as separate clusters (Figure 3); however, these features were not clear in poorly differentiated tumors (PD). Based on cytomorphology alone, 6/7 cases could be diagnosed provisionally as HCC and confirmed later on ICC; the remaining case was provisionally diagnosed as PDHCC or PD metastatic AC, as it did not have the classical cytological features and was later confirmed on ICC. Cases of HB (n=1) with a provisional diagnosis of small round cell tumor (SRCT) and CC (n=2) were confirmed by ICC and histopathology. Out of 20 cases of MC, one case showed atypical squamous cells admixed with benign hepatocytes. On further evaluation, the patient was found to have a primary in the lung. One case had atypical small cells with nuclear overlapping, smudged chromatin, and a necrotic background, which on ICC and histology proved to be a case of metastatic small cell carcinoma (SmCC) lung. Among the 18 AC cases (NOS), 8 cases could be correctly diagnosed on cytology alone; in the remaining 10 cases a possibility of metastatic PDAC was suggested. All these cases of MC were positive for MOC31 and thus confirmed as MC. In a single case of AC, focal neuroendocrine (NE) morphology was also noticed together with an acinar pattern and was later confirmed by NE markers, i.e. Chromogranin (CG) and Synaptophysin (Syn) in addition to MOC31 on ICC. Hence the final diagnosis was AC with NE differentiation. (Table 3)

**Table 3:** Cases showing staining pattern and grading on immunocytochemistry

**Category** **(No of cases)**	**HepPar-1** **(Staining intensity)**	**CD10** **(pattern)**	**CD34** **(Grade)**	**CK7** **(pattern)**	**CK19** **(pattern)**	**MOC31 (pattern)**
**HCC (n=7)**	1(1+) 2 (2+) 3 (3+)	5 (cn)	1 Gr-0 1 Gr-1 3 Gr-2 1 Gr-3	-	-	-
**CC (n=2)**	-	-	-	2 (c ++)	2 (c ++)	2 (c +)
**HB (n=1)**	1 (1+)	-	-	-	-	-
**Metastasis (n=20)**	-	-	-	2(**c ++**)	3 (**c ++**) 2 (**c+**)	8 (cma ++) 8 (m ++) 2 (c++)

**HCC:** Hepatocellular carcinoma, **HB:** Hepatoblastoma, **CC:** Cholangiocarcinoma, **m:** membranous; **c:** cytoplasmic, **cma:** cytoplasmic with membranous accentuation, **cn:** canalicular, **s:** sinusoidal. ++ (diffuse/strong positivity);+ (focal/weak positivity). **HepPar 1:** 1+ (1-5%), 2+ (5-50%), 3+ (>50%). **Grade (Gr) 0:** no staining, Gr 1: < 25% cells staining, Gr 2: 25-50%, Gr 3: 50-75%, Gr 4: >75%

**HepPar-1**: HepPar-1 showed distinct granular cytoplasmic positivity in 6/7(86%) HCC cases (Figure 1, inset) and HB (n=1); while a single case of PDHCC and all cases of MC (n=20) were negative for HepPar-1.

**CD10: **5/7 (71.2%) cases of HCC showed positivity for CD10 (Figure 1, inset) including the PDHCC which was negative for HepPar-1; the staining pattern was canalicular with long branching and zig-zag linear pattern (n=3) and cytoplasmic stain with membranous accentuation (n=2). None of the CC and MC showed positive staining for CD10.

**CD34:** CD34 positive stain was considered when any cell that stained brown with a dot like, linear, semi-circular/circular pattern, and was clearly separate from an adjacent cell. The counting was carried out in 10 high power fields (HPF) and an average was taken which was further graded as 0-4: 0 (no stain), 1 (< 25% cells stain positive), 2 (25-50% cells stain positive), 3 (50-75% cells stain positive), 4 (>75% cells stain positive). In this study, a positive result was found in 6/7 (86%) cases of HCC; grade-1 in one case (3.37%), grade-2 in 3 cases (10%), grade-3 in 2 cases (6.67%), and no staining in one case (3.37%)**.** None of the CC and MCs showed positive stain. There was no correlation between the grade of tumor and positive ICC.

**CK7: **Total of 4 (16.67%) cases were positive for CK7; 2 were CC that showed strong cytoplasmic reactivity and 2 were MC ovary which also showed strong cytoplasmic immunoreactivity (Figure 2). None of the cases of HCC showed positivity.

**CK19: **A positive result was considered when the tumor cells showed either cytoplasmic staining with membrane staining or paranuclear dot like accentuation. A total of seven cases were positive for CK19; 2 were CC (100%) and 5 (25%) were MC (colonic AC-3, strong cytoplasmic positivity, gastric AC-2, focal cytoplasmic positivity) (Figure 2). None of the HCC’s was positive for CK 19.

**MOC31: **MOC31 was expressed in 18/20 (90%) cases of MC; eight showed cytoplasmic positivity with membranous accentuation (40%), eight showed membranous positivity (40%), and two showed cytoplasmic positivity (10%). CC (n=2), in addition to CK 7 and CK19 positivity, also expressed cytoplasmic positivity for MOC31 (Figure 3). None of the cases of HCC were positive.

A few additional markers apart from the primary panel were used (i.e. CG, Syn, S100) to confirm the metastatic origin of certain cancers like SmCC lung and AC with NE differentiation.

## DISCUSSION

Distinguishing HCC from CC and MC on FNAC of neoplastic hepatic masses has been a diagnostic challenge and it is paramount to differentiate these lesions as the prognosis and treatment approaches are different for all three conditions. The areas of challenge include differentiation of benign reactive hepatocytes from WD HCC, PDHCC from MCs and, CC from HCC or metastases. This difficulty is compounded by limited and or non-representative aspirate, and a hemorrhagic and or necrotic background. In recent years, various ancillary techniques such as ICC have contributed greatly to distinguish these lesions and to overcome the diagnostic pitfalls (1–13). The present study highlights certain cytomorphological features helpful in the diagnosis of HCC, especially in WD and MD HCC such as round to polygonal cells, peri and/centri-sinusoidal pattern of tumour cells with small transgressing capillaries, bile pigment, INI, macronucleoli, variable pleomorphism and atypical stripped nuclei in the background. Features of MC included atypical cells in acinar and or papillary pattern, scattered cells, mucinous background and signet ring cells in some; and most importantly benign hepatocytes in separate clusters. However, in PD tumors (HCC vs MC), these features are not clearly identifiable leading to a diagnostic challenge and pitfall on smears. In such cases, ICC is essential to solve this dispute. Various markers have been studied in the past two decades to evaluate the most specific and sensitive markers to differentiate the primary versus metastatic lesions. The present study evaluated HepPar-1, CD 10, CK7, CK19, CD34, and MOC 31 to differentiate HCC from MC.

HepPar-1 (human hepatocyte Ab) recognizes hepatocyte mitochondrial epitope with granular cytoplasmic staining on ICC. Earlier studies showed high sensitivity of HepPar-1 (90%) in detecting HCC (1,4,9,10). However, despite the high sensitivity, it is not entirely specific for hepatocytes, nor could it discriminate between benign and malignant hepatocytes. Also, some studies have reported negative HepPar-1 in PDHCC, and interestingly positive reactivity in other malignancies with hepatoid differentiation (16). In the present study, sensitivity (86%) and specificity (100%) in detecting HCC with HepPar-1 were observed. Negative staining was noted in PDHCC (n=1) and all cases of MC. The above case of PDHCC posed a diagnostic challenge as initially the provisional diagnosis was PDHCC/PDMC with history and cytomorphology. The dilemma continued as HepPar-1 was negative; however, CD10 was the only marker amongst the panel that was positive and confirmed the diagnosis of PDHCC, and hence proved that a single marker i.e. Hep Par1 does not help the cytopathologist in challenging cases. A case of HB provisionally diagnosed as pediatric SRCT was later confirmed as HB with HepPar-1 positivity that ruled out other SRCTs.

CD10 is a commonly used marker for hematolymphoid neoplasms. The positive stain includes a cytoplasmic, membranous/canalicular pattern. The canalicular pattern has been considered specific for hepatocytic differentiation on tissue sections. Saad et al. used CD10 on FNA smears with satisfactory SN (77%) and SP (100%) (4). The present study also showed comparable results for SN (72%) and SP (100%) in detecting HCCs. It was the only positive marker that was expressed in a case of HCC that was negative for HepPar1 and CD34. Recently, Singha et al. showed canalicular, membranous, and membranous and cytoplasmic staining patterns for CD10 (5). The present study also showed these characteristic staining patterns.

Cui et al. first reported strong expression of CD 34 in sinusoidal vessels in all cases of advanced HCC with a high sensitivity (100%) in detecting HCC (6). The present study showed SN of 86% and SP of 100%. Kakar et al. also showed CD 34 expression in WDHCC in a few cases of adenomatous hyperplasia, but none in cirrhosis; hence, supporting the fact that CD 34 positive sinusoids in HCC may suggest microvessel angiogenesis and tumor cell proliferation due to hepato-carcinogenesis (15). This finding correlates in the present study also, since none of the control cases or MC was positive. Although the assessment of angiogenesis does not provide prognostic information, it might help as a diagnostic marker to differentiate HCC from MC.

MOC31 has been proven a sensitive and specific marker of AC differentiation and thus is used to distinguish AC from mesothelioma both in tissue sections as well as in body fluids. Proca and Porcell et al. showed high sensitivity and specificity (100%) in detecting MC and distinguishing it from HCC (2,7). Luoquan et al. studied MOC31 expression in alcohol-fixed, paraffin-embedded liver FNA-CBs and found a high sensitivity (97%) and specificity (94%) (10). In the present study, MOC 31 helped in detecting MACs to liver with a sensitivity of 90% and a specificity of 100% and ruled out primary malignancy; however, it did not help to differentiate MC vs. CC indicating the primary site of tumor.

Expressions of various cytokeratins (CK) has recently gained popularity to distinguish primary sites of liver metastases (8,11) because malignant cells usually maintain the CK profile of their “cells of origin”. Normally, adult hepatocytes express CK 8 and 18 whereas bile duct epithelium expresses CK 7, 19, 8 and 18. CK19 expression is normally found in hepatic progenitor cells and cholangiocytes but not in adult hepatocytes. CK 19 expression appears relatively specific for CC, though HCC may sometimes exhibit CK19 positivity. The present study included two cases of CC, both expressing CK7 & CK19; while none of the cases of HCC were positive for CK 7 and CK19. Few MC expressed either of these markers but none showed positivity for both.

In conclusion, based on the present study, it is observed that a panel of three markers (HepPar, CD10 and MOC31) would help to differentiate HCC from metastatic AC in conjunction with cytomorphological correlation. Both FNA smears and CB can be used for ICC with similar intensity; however, FNAC may have limitations because of a non-representative sample and or a hemorrhagic necrotic background.

## Conflict of INTEREST

The authors declare no conflict of interest.

## FUNDING

None
